# Population Distribution of the Sagittal Abdominal Diameter (SAD) from a Representative Sample of US Adults: Comparison of SAD, Waist Circumference and Body Mass Index for Identifying Dysglycemia

**DOI:** 10.1371/journal.pone.0108707

**Published:** 2014-10-01

**Authors:** Henry S. Kahn, Qiuping Gu, Kai McKeever Bullard, David S. Freedman, Namanjeet Ahluwalia, Cynthia L. Ogden

**Affiliations:** 1 Division of Diabetes Translation, National Center for Chronic Disease Prevention and Health Promotion, Centers for Disease Control and Prevention, Atlanta, Georgia, United States of America; 2 Division of Health and Nutrition Examination Surveys, National Center for Health Statistics, Centers for Disease Control and Prevention, Hyattsville, Maryland, United States of America; 3 Division of Nutrition, Physical Activity, and Obesity, National Center for Chronic Disease Prevention and Health Promotion, Centers for Disease Control and Prevention, Atlanta, Georgia, United States of America; University of East Anglia, United Kingdom

## Abstract

**Background:**

The sagittal abdominal diameter (SAD) measured in supine position is an alternative adiposity indicator that estimates the quantity of dysfunctional adipose tissue in the visceral depot. However, supine SAD’s distribution and its association with health risk at the population level are unknown. Here we describe standardized measurements of SAD, provide the first, national estimates of the SAD distribution among US adults, and test associations of SAD and other adiposity indicators with prevalent dysglycemia.

**Methods and Findings:**

In the 2011–2012 National Health and Nutrition Examination Survey, supine SAD was measured (“abdominal height”) between arms of a sliding-beam caliper at the level of the iliac crests. From 4817 non-pregnant adults (age ≥20; response rate 88%) we used sample weights to estimate SAD’s population distribution by sex and age groups. SAD’s population mean was 22.5 cm [95% confidence interval 22.2–22.8]; median was 21.9 cm [21.6–22.4]. The mean and median values of SAD were greater for men than women. For the subpopulation without diagnosed diabetes, we compared the abilities of SAD, waist circumference (WC), and body mass index (BMI, kg/m^2^) to identify prevalent dysglycemia (HbA1c ≥5.7%). For age-adjusted, logistic-regression models in which sex-specific quartiles of SAD were considered simultaneously with quartiles of either WC or BMI, only SAD quartiles 3 (p<0.05 *vs* quartile 1) and 4 (p<0.001 *vs* quartile 1) remained associated with increased dysglycemia. Based on continuous adiposity indicators, analyses of the area under the receiver operating characteristic curve (AUC) indicated that the dysglycemia model fit for SAD (age-adjusted) was 0.734 for men (greater than the AUC for WC, p<0.001) and 0.764 for women (greater than the AUC for WC or BMI, p<0.001).

**Conclusions:**

Measured inexpensively by bedside caliper, SAD was associated with dysglycemia independently of WC or BMI. Standardized SAD measurements may enhance assessment of dysfunctional adiposity.

## Introduction

The body mass index (BMI, weight/height^2^) is recommended for clinical and epidemiological assessments of human adiposity [1], [2], but BMI cannot distinguish between lean mass and depots of adipose tissue (AT). Dependence on the categorical BMI has sometimes misclassified health risk, leading commentators to call for the exploration of alternative, low-cost, adiposity metrics [3]. A candidate alternative indicator is the sagittal abdominal diameter (SAD) which, when measured externally in the supine position (“abdominal height”), estimates the volume of visceral (intra-abdominal) AT [4], [5]. As demonstrated by expensive imaging technologies, it is primarily the visceral depot of AT (as opposed to subcutaneous depots) that correlates with cardiometabolic risk [6]–[8]. Associations have been found between SAD and chronic-disease risk factors or outcomes, but these reports depended on selected research populations and employed varying methods and positions for measuring SAD [9]–[16]. Wider use of the SAD would benefit from a standardized measurement protocol and the availability of SAD normative reference values.

This paper describes a simple, inexpensive protocol for SAD measurement and estimates the distribution of SAD values in the US adult population examined during 2011–2012. It also demonstrates how the use of SAD measurements could improve upon BMI or waist circumference (WC) for the recognition of impaired glucose regulation (“dysglycemia”).

## Methods

### Participants and their clinical measurements

The National Health and Nutrition Examination Survey (NHANES) is a nationally representative, cross-sectional survey of the resident civilian, non-institutionalized, US population. Participants in NHANES underwent home interviews followed by standardized anthropometric and laboratory assessments in mobile examination centers. The complex, multistage-probability, sampling design of NHANES requires sample weights for each participant so that characteristics of the US population can be estimated. In the 2011–2012 NHANES, of 5560 interviewed adults (≥20 years old), 5319 were examined, and 4817 had SAD measurement data. Since pregnant women (n = 57) were not eligible for SAD measurement, the participation rate for SAD was 88% among non-pregnant interviewees. A general description of NHANES has been published elsewhere [17].

**Figure 1:**
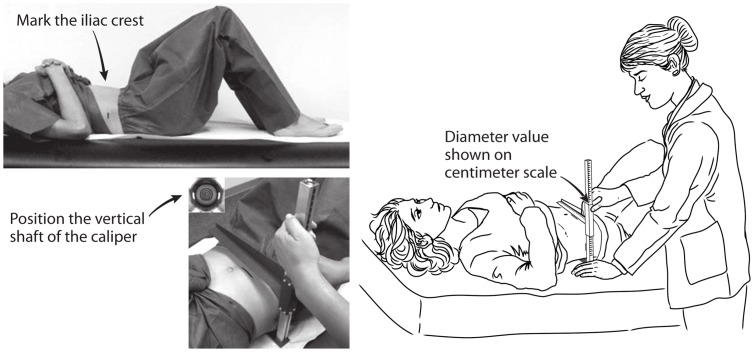
Measurement of the sagittal abdominal diameter by use of a sliding-beam caliper in NHANES, 2011–2012.

The SAD was measured using a sliding-beam, abdominal caliper (Holtain, Ltd, Wales, UK). Supine participants rested on a lightly padded exam table with their hips in a relaxed, flexed position as the examiner marked the level of their iliac crests with a wax pencil. The lower arm of the caliper was then inserted under the small of the back, and the upper arm was raised above the belly in alignment with the transverse pencil mark (Figure 1). After confirming that the caliper shaft was vertical, the examiner asked the participant to inhale gently, slowly let the air out, and then pause (“… rest,… relax…”). The examiner then slid down the caliper’s upper arm, letting it lightly touch the abdomen but without compressing it. The SAD value was read directly from the centimeter scale on the caliper shaft and recorded to the nearest 0.1 cm [18]. Then, after raising the caliper’s upper arm and repeating breathing instructions, a second SAD measurement was recorded. If the two SAD values differed by >0.5 cm, third and fourth measurements were obtained. For this report we defined each participant’s SAD as the mean of 2 initial measurements or of up to 4 measurements as specified in the online, analytic notes from NHANES [19]. Weight, height and a standing-position WC were measured by established methods [18].

Within our analytic sample we identified adults with diagnosed diabetes by their affirmative answer to the question “have you ever been told by a doctor or other health professional that you have diabetes or sugar diabetes?” For those without diagnosed diabetes we defined categorical dysglycemia by a glycated hemoglobin (HbA1c) concentration ≥5.7% (≥39 mmol/mol). This is a common threshold value that points to an increased risk of cardiovascular disease [20] as well as to “prediabetes” or undiagnosed diabetes [21]. Assays of HbA1c in NHANES were performed on whole-blood hemolysate presented to a high-performance liquid chromatography column (Fairview Medical Center Laboratory, University of Minnesota, Minneapolis).

### Ethics Statement

The NHANES protocol was approved by the Research Ethics Review Board of the National Center for Health Statistics; participants provided informed consent.

### Statistical analyses

All analyses accounted for the sampling weights and sample design using SAS (release 9.3 [SAS Institute Inc., Cary, NC], SUDAAN (release 11.1) [RTI International, Research Triangle Park, NC]) or the ‘survey’ package in R [22], [23]. We estimated the distribution of SAD values in 2011–2012 among US adults overall and by sex and age group (20–34, 35–49, 50–64, and 65+ years). The means, quartiles, and their corresponding Wald 95% confidence intervals were calculated using the DESCRIPT procedure of SUDAAN.

For the subpopulation not diagnosed with diabetes, we then assessed the utility of SAD compared to other adiposity indicators (WC or BMI) for identifying prevalent dysglycemia. Our first approach examined the relation of sex-specific quartiles of SAD, WC and BMI to this outcome of interest. Predictive margins from age-adjusted logistic regression models were estimated to provide prevalence ratios (PRs) relative to the lowest quartile; each model’s goodness of fit was estimated as R^2^ (Cox & Snell method). We examined ordinal quartiles for each adiposity indicator individually, as well as the independent effect of SAD quartiles in models that also included either BMI quartiles or WC quartiles.

The three adiposity indicators were highly correlated with each other, and collinearity might complicate interpretation of the individual regression coefficients in models that simultaneously contained SAD and another adiposity indicator. Therefore, we also calculated receiver operator characteristic curves for each indicator and compared the areas under these curves (AUCs) as indices of fit for the various models. These sex-specific logistic regression models included age and an adiposity indicator modeled as continuous variables using natural splines with three knots to allow for non-linearity. They also included a term for sex when the sample included both men and women. Each model’s goodness of fit was estimated as R^2^ (Nagelkerke method). We assessed the difference in the AUCs between models using jackknife resampling [24] with the ‘withReplicates’ function in R [22] to estimate the standard error of the difference between models.

## Results

SAD means and selected percentile values for US adults are presented in Table 1. These estimates for calendar years 2011–2012 were derived from 4817 examined adults (irrespective of metabolic status or other anthropometry; not pregnant) who represented the US non-institutionalized, civilian, population of approximately 224 million at age ≥20 years. The mean and median values of SAD were greater for men than women. In both sexes the means and medians of SAD increased with age at least through 64 years.

**Table 1:** Population mean and median (50^th^ percentile) values with selected percentiles of the sagittal abdominal diameter in US adults, from NHANES 2011–2012.

				Population Percentiles, *cm*				
Sex	Age, y	Sample N	Mean (95% CI), *cm*	5th	25th	50th (95% CI)	75th	95th
Both	**≥20**	**4817**	22.5 (22.2−22.8)	16.4	19.2	21.9 (21.6−22.4)	25.2	30.5
	20–34	1292	21.0 (20.6−21.5)	15.9	17.8	20.1 (19.7−20.8)	23.4	29.1
	35–49	1240	22.5 (22.0−23.0)	16.4	19.4	21.9 (21.3−22.5)	25.0	30.8
	50–64	1311	23.5 (23.0−24.0)	17.3	20.3	23.1 (22.4−23.7)	26.1	31.9
	≥65	974	23.4 (22.9−23.8)*	17.5	20.5	23.2 (22.6–23.5)	25.9	30.4
Men	**≥20**	**2450**	23.2 (22.8–23.6)	17.3	20.1	22.7 (22.2–23.2)	25.8	31.2
	20–34	696	21.5 (20.9–22.0)	16.3	18.3	20.8 (19.9–21.4)	23.7	29.1
	35–49	610	23.3 (22.9–23.7)	17.9	20.4	22.8 (22.3–23.4)	25.3	31.1
	50–64	638	24.4 (23.8–24.9)	17.9	21.3	23.7 (23.2–24.6)	26.9	32.4
	≥65	506	24.3 (23.7–24.9)*	18.6	21.7	23.8 (23.4–24.5)	26.7	31.6
Women	**≥20**	**2367**	21.8 (21.5–22.1)†	15.9	18.5	21.1 (20.8–21.5)	24.7	30.0
	20–34	596	20.6 (20.1–21.0)‡	15.3	17.1	19.6 (18.8–20.0)	22.8	29.1
	35–49	630	21.6 (21.0–22.2)†	15.7	18.2	20.8 (20.2–21.8)	24.4	30.2
	50–64	673	22.7 (22.1–23.3)†	16.9	19.4	21.8 (21.2–23.5)	25.3	30.6
	≥65	468	22.5 (21.9–23.2)* †	16.9	19.3	22.3 (21.3–23.2)	25.4	29.2

* p<0.001 for age trend.

† p<0.001 compared to men.

‡ p<0.01 compared to men.

**Figure 2:**
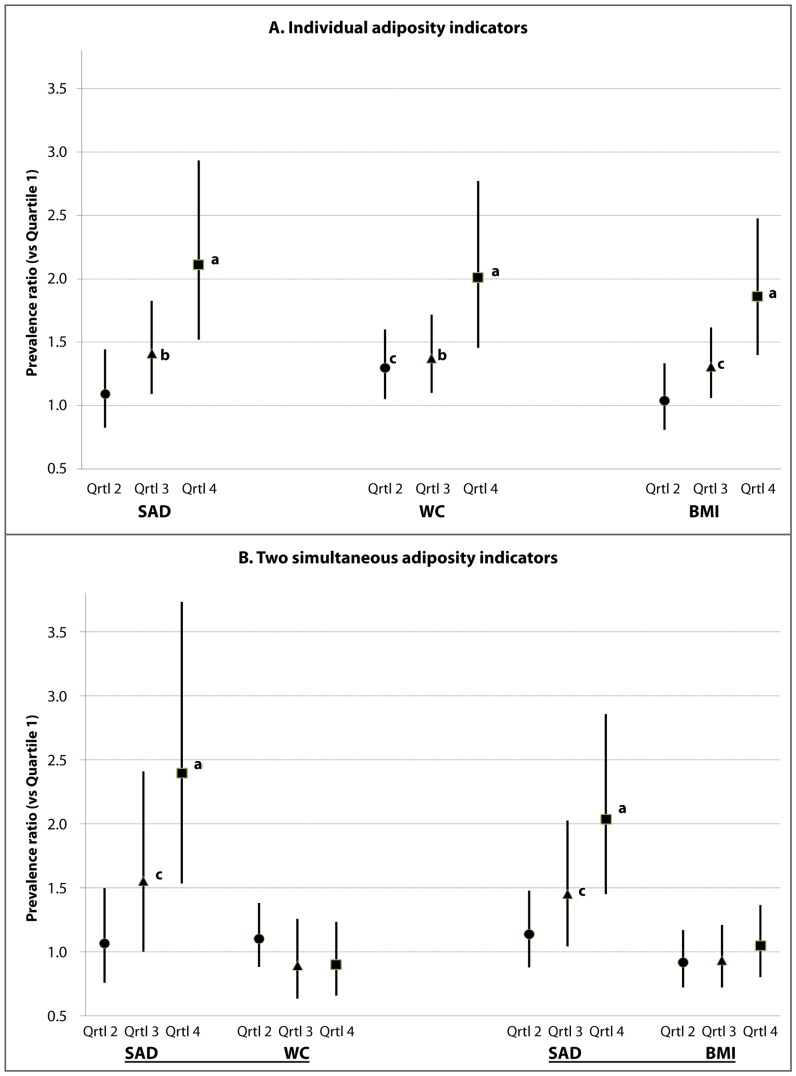
Panel A: Dysglycemia prevalence ratios by quartiles of sagittal abdominal diameter (SAD), waist circumference (WC) or body mass index (BMI). Panel B: Prevalence ratios when SAD is considered simultaneously with WC (left side) or with BMI (right side). *In age-adjusted models, the relative prevalence of dysglycemia (HbA1c ≥5.7% [≥39 mmol/mol]) is displayed in association with the second (circle), third (triangle), and fourth (square) quartiles (with reference to first quartile) of each indicator. Error bars indicate 95% confidence intervals. ^a^ p<0.001; ^b^ p<0.01; ^c^ p<0.05.*

Among adults without a diabetes diagnosis who were evaluated for prevalent dysglycemia, the analytic subpopulation (subsample n = 4037; excluding participants without information on HbA1c, WC, or BMI) included dysglycemic persons with prediabetes or undiagnosed diabetes. For our initial assessment of how dysglycemia would be identified by the 3 adiposity indicators, the sex-specific quartile cutoffs for SAD, WC and BMI are shown in Table 2. The dimensions describing abdominal size (SAD and WC) had cutoff values for men consistently larger than those for women. However, for the indicator of generalized relative weight (BMI), there was no consistent sex distinction for the quartile cutoff values. The overall crude prevalence of dysglycemia in this subpopulation was 26.4%, similar for men (25.9% [95% confidence interval 23.5–28.3]) and women (26.8% [23.8–29.9]). The crude dysglycemia prevalence estimates across the ordinal quartiles demonstrated an increasing trend (p<0.01) of each adiposity indicator (Table 3). In age-adjusted logistic models, the ordinal quartiles of each adiposity indicator were likewise associated with an increasing prevalence of dysglycemia (Figure 2, panel A). The explained variations in dysglycemia (multiple R^2^) for these quartile-based models were 0.133 for SAD, 0.123 for WC, and 0.125 for BMI. When SAD quartiles were simultaneously considered with quartiles of WC in the model, SAD quartile 3 (PR 1.55; p<0.05) and quartile 4 (PR 2.39; p<0.001) remained significantly different from SAD quartile 1 (Figure 2, panel B). When quartiles of SAD were simultaneously considered with quartiles of BMI in the same model, dysglycemia prevalence of SAD quartile 3 (PR 1.45; p<0.05 *vs* quartile 1) and quartile 4 (PR 2.03; p<0.001 *vs* quartile 1) likewise remained significantly elevated. However, for both of these models that tested simultaneous indicators, the competing quartiles 3 and 4 of BMI or WC were not significantly associated with dysglycemia.

**Table 2:** Subpopulation quartile cutoffs of adiposity indicators in US adults ages ≥20 years without diagnosed diabetes, estimated from NHANES 2011–2012.

			Quartile cutoffs (95% confidence interval)		
Indicator	Sex	Subsample n	25^th^ percentile	50^th^ percentile	75^th^ percentile
SAD, *cm*	**Total**	**4,037**	19.0 (18.6–19.4)	21.6 (21.3–22.0)	24.8 (24.2–25.2)
	Men	2,035	19.9 (19.5–20.4)	22.4 (22.0–22.9)	25.4 (24.8–25.9)
	Women	2,002	18.3 (17.9–18.7)	20.8 (20.3–21.3)	24.0 (23.5–24.6)
WC, *cm*	**Total**	**4,037**	86.0 (84.6–87.9)	96.0 (95.1–97.3)	106.5 (105.4–107.8)
	Men	2,035	89.6 (87.5–91.7)	99.0 (97.5–100.6)	108.8 (107.7–110.5)
	Women	2,002	82.9 (81.6–84.6)	93.4 (91.8–94.9)	104.1 (102.2–105.6)
BMI, *kg/m^2^*	**Total**	**4,037**	23.8 (23.5–24.4)	27.2 (26.8–27.7)	31.3 (30.8–31.9)
	Men	2,035	24.3 (23.8–24.8)	27.5 (27.0–27.9)	31.0 (30.5–31.7)
	Women	2,002	23.4 (23.0–23.9)	26.9 (26.3–27.5)	31.8 (31.0–32.5)

**Table 3:** Crude prevalence (%) of dysglycemia by quartiles of adiposity indicators in US adults ages ≥20 years without diagnosed diabetes, estimated from NHANES 2011–2012.

		Crude prevalence (95% confidence interval) of dysglycemia, *percentage*			
Indicator	Sex	1^st^ quartile	2^nd^ quartile	3^rd^ quartile	4^th^ quartile
SAD	**Total**	14.4 (10.8–18.9)	19.9 (16.3–24.0)	28.9 (25.6–32.5)	42.0 (37.4–46.7) ^∥^
	Men	13.8 (10.1–18.7)	21.5 (16.4–27.7)	26.5 (21.2–32.5)	41.4 (36.1–46.9) ^∥^
	Women	14.9 (10.4–20.7)	18.3 (14.1–23.3)	31.3 (25.8–37.3)	42.5 (35.4–50.0) ^∥^
WC	**Total**	14.1 (10.7–18.4)	23.9 (21.1–27.0)	28.1 (23.9–32.7)	39.1 (34.6–43.7) ^∥^
	Men	14.4 (10.5–19.4)	22.7 (19.4–26.5)	26.4 (21.5–32.0)	39.6 (33.9–45.7) ^∥^
	Women	13.8 (10.0–18.8)	25.0 (19.4–31.6)	29.7 (24.0–36.1)	38.5 (33.0–44.3) §
BMI	**Total**	18.3 (14.4–22.9)	21.7 (17.3–26.7)	28.4 (24.6–32.6)	36.7 (32.0–41.7) ^∥^
	Men	19.6 (14.6–25.7)	21.6 (17.1–26.7)	26.9 (22.1–32.2)	35.1 (29.0–41.7) ^∥^
	Women	17.0 (12.0–23.5)	21.7 (16.1–28.7)	29.9 (24.7–35.8)	38.3 (32.0–45.1) ^∥^

∥ p<0.001 for quartile trend.

§ p<0.01 for quartile trend.

When our age-adjusted models with competing quartiles (“SAD *vs* WC” or “SAD *vs* BMI”) were restricted to either sex, SAD quartiles 3 and 4 again provided elevated point estimates although their confidence intervals did not always exclude one. For men (subsample n = 2035), when competing with WC quartiles, the SAD quartile 3 had PR 1.57 [0.89–2.76] and SAD quartile 4 had PR 2.31 [1.36–3.92]; when SAD competed with BMI quartiles, the men’s SAD quartile 3 had PR 1.57 [0.92–2.68] and SAD quartile 4 had PR 2.28 [1.49–3.49]. For women (subsample n = 2002), when competing with WC quartiles, the women’s SAD quartile 3 had PR 1.60 [1.00–2.56] and SAD quartile 4 had PR 2.52 [1.56–4.06]; when SAD competed with BMI quartiles, the SAD quartile 3 had PR 1.35 [0.84–2.16] and SAD quartile 4 had PR 1.83 [1.15–2.92]. In these sex-specific models all the quartiles of BMI or WC had weaker, non-significant associations with dysglycemia (PRs <1.21).

**Figure 3:**
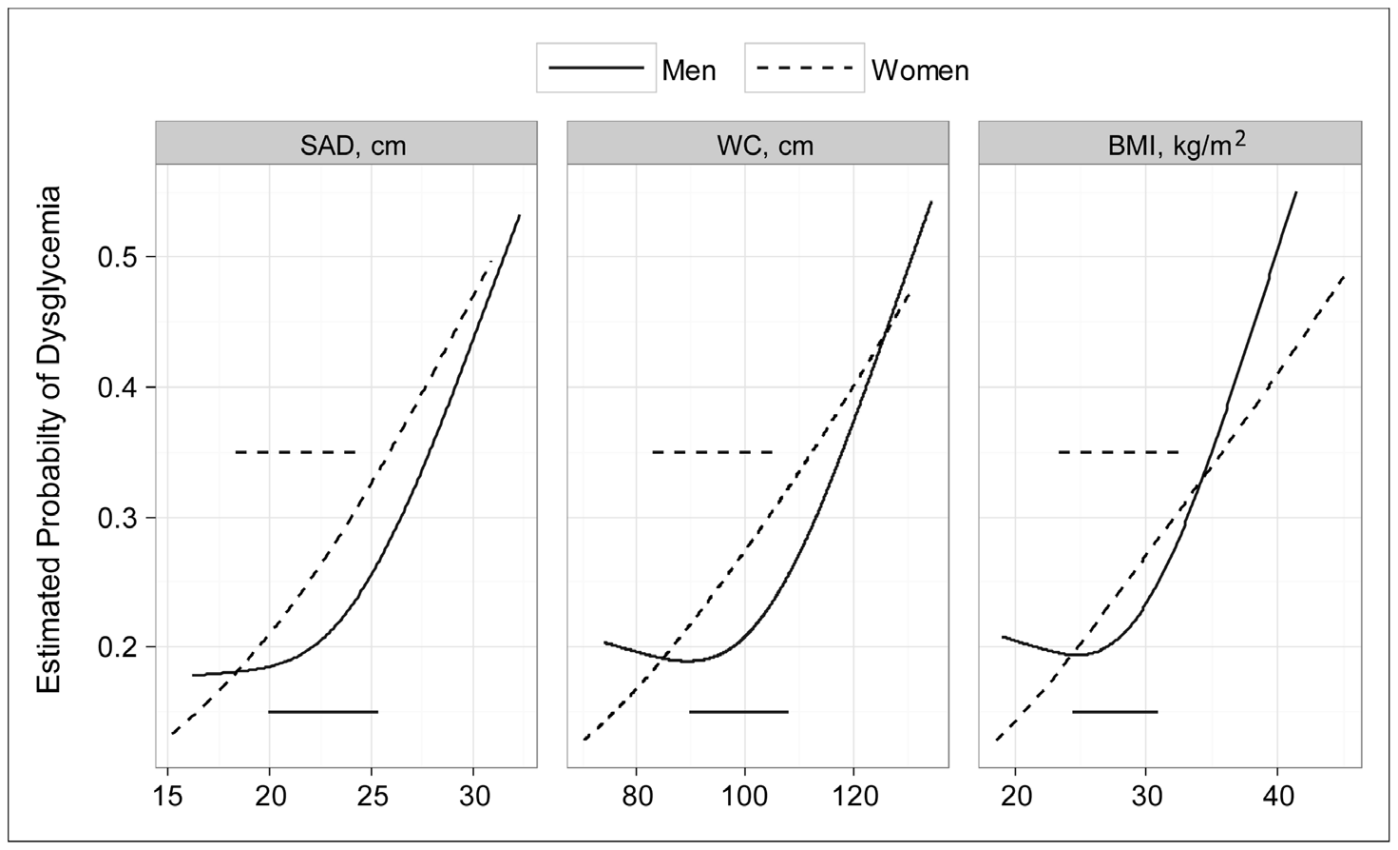
Probability of prevalent dysglycemia estimated by continuous sagittal abdominal diameter, waist circumference or body mass index. *In these age-adjusted plots prepared by restricted cubic splines, the horizontal lines represent the interquartile range (p25 to p75) in the sex-specific population distributions of each adiposity indicator*.

In the assessment of how well the continuous adiposity indicators identified dysglycemia (our second approach), the competing models adjusted for age and sex tended to confirm that continuous SAD explained a greater proportion of dysglycemia than continuous WC or BMI. Multiple R^2^ values for these continuous models were 0.201 for SAD, 0.195 for WC, and 0.198 for BMI. The differences between these AUCs were non-significant for the models in which both sexes were analyzed together (Table 4), but sex interactions were found for all three adiposity indicators. In sex-stratified analyses the men’s AUC for SAD was greater (p<0.001) than the AUC for WC (but not greater than the AUC for BMI); the women’s SAD area was greater (p<0.001) than the AUC for either WC or BMI. In sex-specific, age-adjusted curves we found for each of the adiposity indicators that the relationship with dysglycemia was curvilinear for men (J-shaped) but nearly linear for women (Figure 3).

**Table 4:** Areas under the curve (AUCs) of the receiver operating characteristic (ROC) for identification of dysglycemia by an adiposity indicator adjusted for age; comparisons of SAD with WC or BMI.

			AUCs (areas under the ROC curve)			p-value for difference in areas	
Population		Subsample n	SAD	WC	BMI	SAD vs. WC	SAD vs. BMI
Total*		**4037**	0.748	0.741	0.744	NS	NS
Sex	Men	2035	0.734	0.728	0.732	<0.001	NS
	Women	2002	0.764	0.757	0.758	<0.001	<0.001

* Model includes adjustment for sex.

NS, p>0.10.

## Discussion

Adult SAD measurements obtained in NHANES 2011–2012 demonstrate the feasibility and utility of assessing abdominal adiposity with a portable, sliding-beam caliper. Identical or very similar anthropometric protocols have been used previously in studies of diabetes, incident coronary heart disease and several cardiometabolic risk factors among selected adults [11]–[14], [16], [25]–[29] and in a Finnish national survey of persons ≥30 years [30]. The choice of the iliac crests (approximating lumbar interspace L4–L5) as the most effective measurement landmark has been recommended by reports that also evaluated alternative sites such as the L3–L4 interspace, the umbilical level, or the highest point on the abdomen [31]–[33].

The historical rationale for measuring SAD has been the presumption that variation in this simple dimension would reflect increases in the amount primarily of visceral AT. An early proponent of the SAD pointed out that visceral AT would tend to ‘pump up’ the abdomen in the sagittal direction of supine subjects [34], and later investigators confirmed that the surrounding subcutaneous AT would tend to flow out at the flanks [35]. Recent advances in AT imaging, however, have demonstrated that subcutaneous AT contains distinct *deep* and *superficial* sub-compartments, each with its own histologic and physiologic characteristics. Deep abdominal subcutaneous AT may be located primarily near the anatomic midline (as inferred from cross-sectional abdominal images). This deep subcutaneous sub-compartment is associated, notably among men, with increased levels of circulating HbA1c [36] and other cardiometabolic risk variables [37]. Superficial abdominal subcutaneous AT is relatively more prominent at the sides of the abdomen, and its physiologic correlates are relatively benign. If the SAD incorporates primarily the deep (midline) subcutaneous AT but less of the superficial (lateral) AT, this might explain why prior research reported the SAD, when compared to the area of visceral AT alone, was more strongly associated with the metabolic syndrome and other cardiometabolic risk variables [14], [38], [39].

The distinction between deep and superficial subcutaneous AT may help to explain also why men, but not women, have a J-shaped relation of adiposity to dysglycemia prevalence (Figure 3). A deficit of superficial subcutaneous AT may be considered a marker of metabolic dysfunction since adipocytes in this sub-compartment are capable of safely storing energy during positive caloric balance. Compared to women, men have lesser amounts of superficial subcutaneous AT in the abdominal region [36], [37]. Some men with low levels of generalized adiposity may have so little superficial subcutaneous AT that any net excess of energy intake will result in an overflow to less benign AT depots or to ectopic sites such as the liver, skeletal muscle or pancreas. Others have previously commented on metabolic dysfunctions that occur when subcutaneous AT, irrespective of its sub-compartments, fails to expand sufficiently in response to metabolic overload [40], [41]. Given that the anthropometric methods of NHANES cannot directly distinguish between the deep and superficial components of subcutaneous AT, this speculative explanation of the J-shaped relationship to dysglycemia cannot be tested in our dataset.

Type 2 diabetes has been related to adiposity phenotypes that have an increased volume of visceral AT or elevations of hepatic fat content [6], [7], [42]. An enlarged visceral adipose depot and hepatic steatosis both represent forms of ectopic fat deposition. Since the SAD is associated with visceral AT volume [34] it is reasonable to expect that this easily measured external dimension would be associated also with dysglycemia and with an increased risk of diabetes. Direct assessments of hepatic fat content could likewise provide correlations with dysglycemia and cardiometabolic risk, but such assessments depend on liver biopsy or technologies (e.g., multi-slice magnetic resonance or tomographic imaging, magnetic resonance spectroscopy) that carry substantial costs in time, money, and possibly radiation.

Our finding that SAD was associated with dysglycemia in the general US adult population, independently of age and of WC or BMI, confirms smaller studies of SAD restricted to obese adults [13], [28]. Hyperinsulinemia, a marker of insulin resistance, has likewise been associated with SAD among young adults [12] and among older men without diabetes [13]. A prospective comparison from Finland of four adiposity indicators measured at ages ≥30 years has reported recently that the co-occurrence of high BMI and high SAD, but not high WC or high waist-to-hip ratio, was associated with the highest incidence of type 2 diabetes [30].

The absence of prospective, follow-up information is a major limitation of our study. Current survey data from NHANES are necessarily cross-sectional, although some earlier waves of NHANES examinations have been followed by re-contact [43] or mortality reviews [44]. Measurements of SAD within NHANES did not begin, however, until 2011. The Finnish national survey mentioned above was conducted in 2000–2001, and it employed an SAD protocol nearly identical to that used by NHANES. Smaller studies based on selected populations have reported prospectively on mortality [45]–[47] and incident dementia [48] in association with the SAD, but their anthropometric protocols differed substantially from that of SAD in NHANES. With regard to our participants who reported not having diabetes, another possible limitation of our study is the dependence on an assay of HbA1c to define the metabolic outcome of interest. However, the common limitations of HbA1c interpretation [49] are likely to be minimized in our analyses since all HbA1c assays for NHANES were performed by a single, highly standardized laboratory.

Consistent with physiologic and anatomic principles, the SAD stands as a credible alternative to the conventional WC or BMI for the clinical assessment of adiposity. As validated in this nationally representative sample, SAD could inexpensively augment the understanding of abdominal AT and its associated health risks. The public-use NHANES data will provide opportunities to test cross-sectional associations between SAD and many biomarkers or clinical conditions. Future studies employing a prospective design could expand on these findings and explore the associations of this adiposity indicator with medical outcomes and mortality.
